# A simple and effective heat induced antigen retrieval method

**DOI:** 10.1016/j.mex.2016.04.001

**Published:** 2016-04-08

**Authors:** Vinod K.R., Denny Jones, Venkatesha Udupa

**Affiliations:** Department of Toxicology, Glenmark Research Centre, Navi Mumbai 400709, India

**Keywords:** Modified HIER method, Antigen retrieval, Immunostaining, Tissue damage, New method

## Abstract

In this paper, we describe an additional step to the standard method of heat induced antigen retrieval to improve the detection of antibody staining of formalin fixed paraffin embedded tissue sections. Direct heating of tissues in buffer is an efficient epitope retrieval method but often results in the damage or loss of tissues. In this modified method, before keeping in buffer for heating, we overlapped the tissue on the slide with a plain slide by clipping one end using a normal paperclip, keeping a minimum gap between the slides. Tissues heated in this way in buffer had following advantages over normal heat treatment for epitope retrieval.

• Tissues were intact even at high temperatures which improved the quality of staining by preventing fold, damage or detachment of tissues from the slides.
• The method is very safe and economical compared to the methods using microwave or pressure cooker.
• This simple method also appears to be very effective and less time consuming compared to the existing methods.

## Methodology background

Antigen retrieval is an important step in immunohistochemistry staining. The simple technique of boiling formalin-fixed paraffin embedded (FFPE) tissue sections in water has played a major role in extending the reach and use of immunohistochemistry [1]. However, direct boiling of tissues in buffer often results in the damage of tissues or falling off tissues from the slide. Several reasons such as insufficient fixation, improper sectioning and drying, poor adherence and even uncleaned slides have been reported to contribute to the issue of falling off or detachment of tissues from slide [2], [3], [4], [5]. Even after taking care of above factors, the sections often tend to detach from adherent coated slides while boiling. In this paper, we present an additional step to the standard heat induced epitome retrieval (HIER) of tissues by which the damage or detachment of tissue from the slide while boiling can be prevented.

## Tissue collection and preparation

Tissues collected from male Sprague-Dawley rats aged 8–12 weeks used for this study. After fixation in 10% neutral buffered formalin, paraffin embedded tissues were cut at 4–5 μm and transferred to HistoGrip coated glass slides. Tissue sections were dried on a slide warmer at 60 °C prior to immunostaining.

## Antigen retrieval method

Deparaffinised and rehydrated slides were kept in a solution of sodium citrate (pH 6.0) or Tris/EDTA (pH 9.0), once the temperature has reached 95 °C in a water bath.

**Fig. 1:**
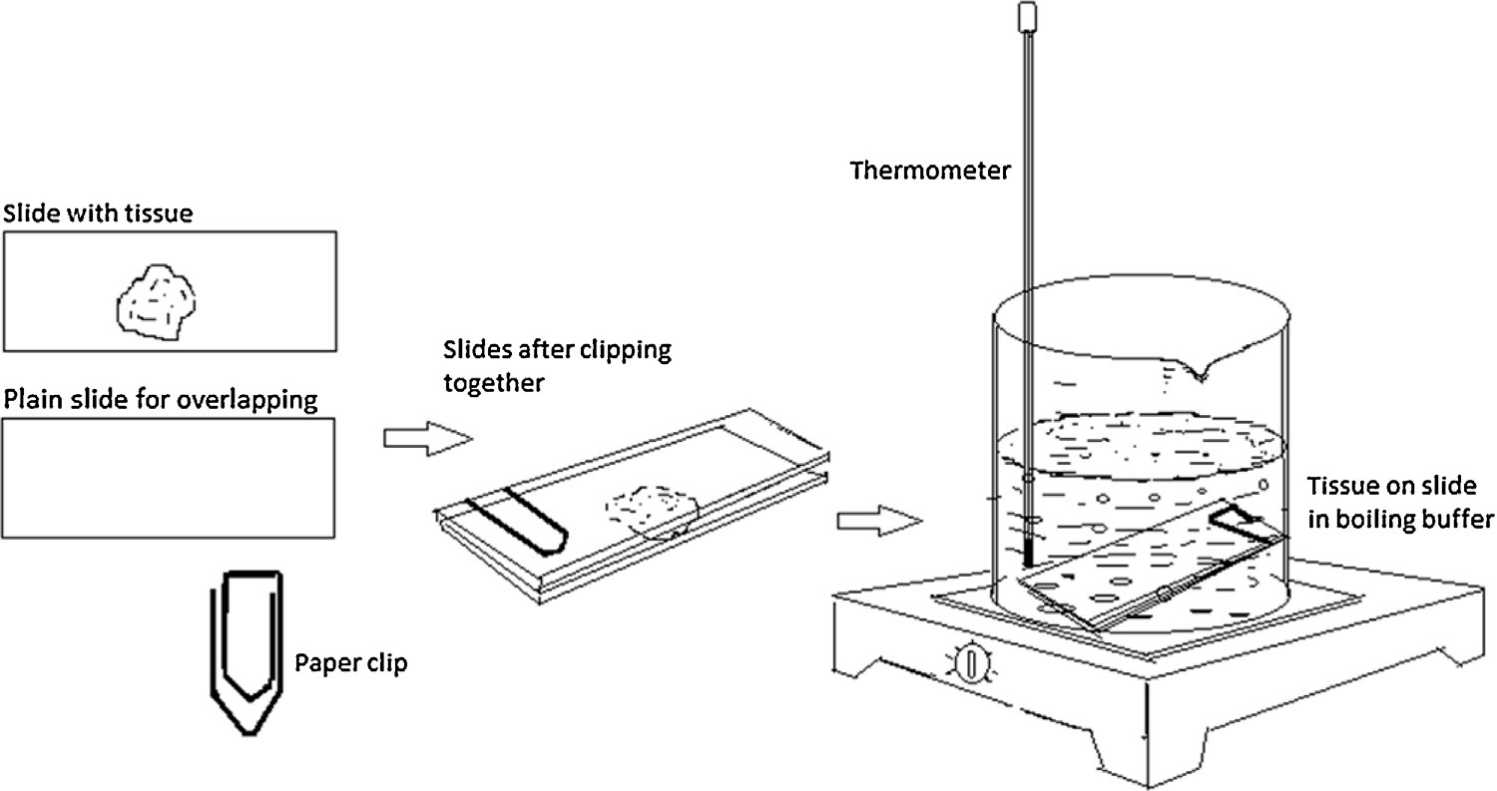
Additional step in antigen retrieval.

Additional step (Fig. 1) in antigen retrieval method is described below.

Before keeping the slides in buffer for heating in a temperature controlled water bath or in a beaker on hotplate, the tissue bearing slide was overlapped with another plain slide by clipping one end using a normal paperclip (U clip of 2″). Clipping is done only at one end in such a way that the other end gets slightly widened allowing the buffer to go to the tissue and the tissue does not get jammed between the slides. Also one end is slightly shifted laterally (clockwise) in order to make it easier to remove the overlapping slide after the heat treatment. Each tissue slide was paired with a plain slide separately in this way before keeping in buffer for boiling. Tissues were boiled for 15 min at temperature ranging from 95 to 100 °C. Slides were then taken out of water bath and allowed to cool in a vessel of tap water for 10 min. The clips were removed safely when the tissues were in cold water.

## Immunostaining

Immunoperoxidase staining was performed as per the standard Avidin-Biotin Complex (ABC) method. Precisely, primary antibody was incubated for 1 h and secondary antibody for 30 min to 1 h at room temperature. Biotinylated enzyme (HRP) was preincubated with free avidin for about 15 min and an aliquot of this solution was added to the tissue sample for further incubation for 30 min. Slides were stained with diaminobenzidine (DAB) chromogen and counterstained with hematoxylin.

## Method validation

**Fig. 2:**
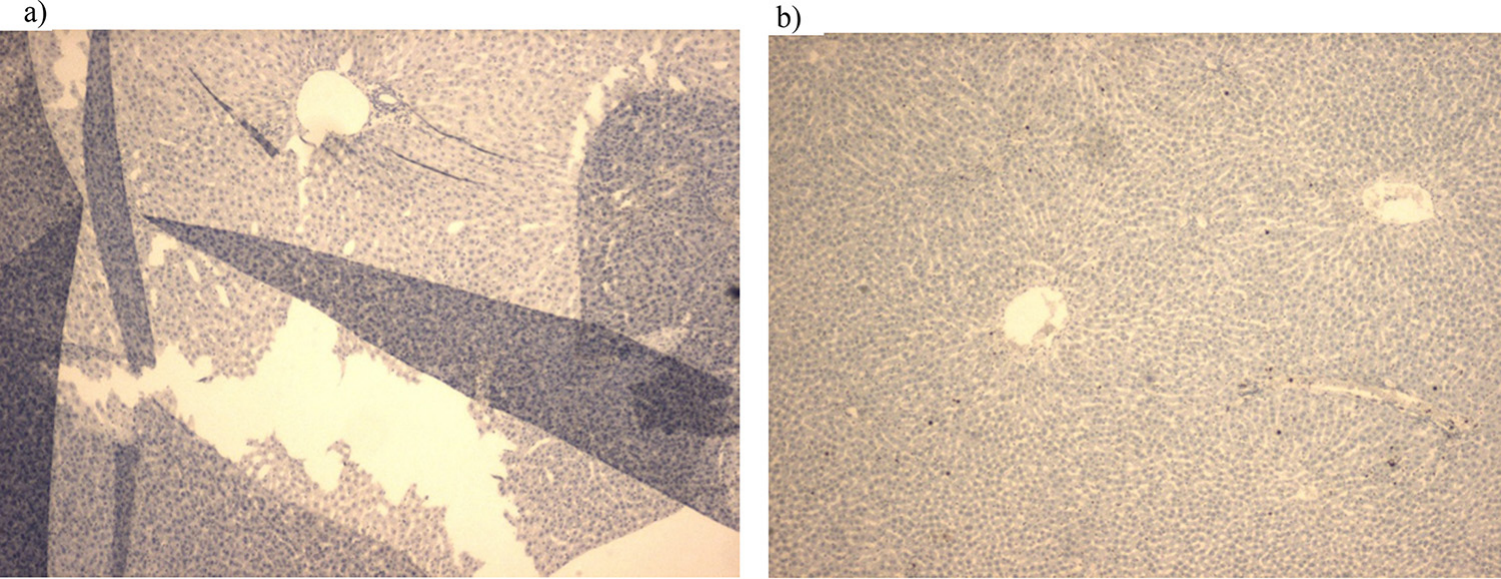
Difference in tissue morphology after employing the modified method a) without clippingb) with clipping.

**Fig. 3:**
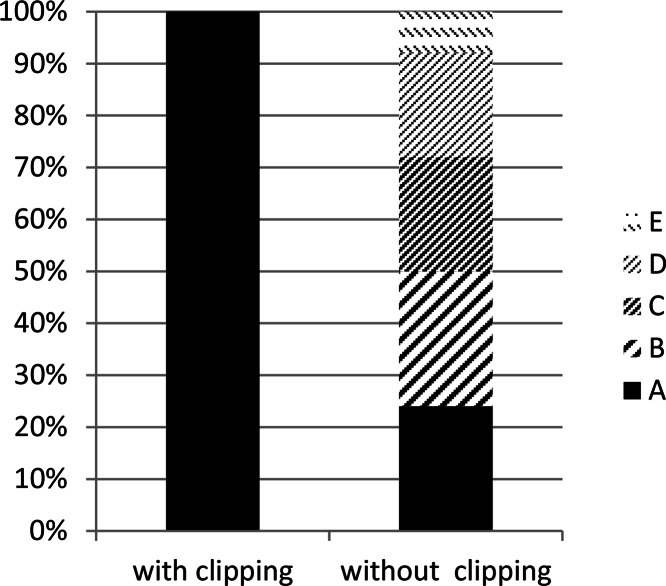
A comparison of tissue morphology after directly boiling in buffer (1) with clipping and (2) without clipping (percentage of instances of tissue damage calculated from a total of 50 samples, comprising 10 samples from heart, kidney, liver, thymus and intestine). A – No damage, B – Slightly damaged, C – Moderately damaged, D – Severely damaged, E – Completely lost.

In the present study, we have performed IHC staining with several antibodies with and without the additional step in the standard protocol. We found that the direct boiling without covering slides often resulted in loss of tissue from the slide. Additional step has always given the best results. Tissues were intact on the slide for further processing which improved the quality of staining by preventing damage or detachment of tissues from the slides. The comparisons of tissue morphology after directly boiling in buffer with and without slide clipping are given in Fig. 2 (qualitative) and Fig. 3 (quantitative). This simple additional step is also safe and economical. Protease digestion was the first method used to counteract the antigen masking effects of formalin fixation. However, since the advent of heat induced epitope retrieval (HIER) techniques, proteases play a much smaller role in most IHC laboratories [6]. Microwave ovens are also used for HIER. However, normal microwave ovens are notorious for having hot and cold spots, while laboratory microwaves are very expensive. Using pressure cooker is yet another alternative, however, not very safe and feasible to handle as a normal water bath in a laboratory.

In this modified method, we boiled the sections in sodium citrate at pH 6 or TrisEDTA at pH 9 buffer (with 0.05% Tween 20) at a temperature ranging from 95 °C to 100 °C for 15 min. Optimal antigen retrieval time may vary as per the antibody specification; however, we have achieved the best results between 10–20 min for several antibodies such as CD 45, CYP 3 A, PCNA etc. Tissues can be boiled in this way even more than 30 min without any damage or loss from the slide. Slides can be kept in a beaker or water bath directly without a slide rack as there is no chance of sticking the tissues from different slides together; the tissue on each slide is protected by a plain slide. Detachment of sections from the slide may be due to vigorous movement of water molecules resulting from the convection current while boiling. When overlapping with another slide with a little space between the slides, it creates only a minimum pressure on the tissue, thus protecting it from falling off the slide.

**Fig. 4:**
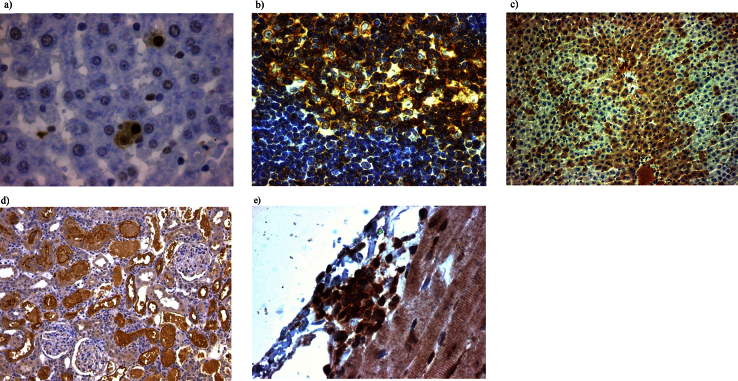
Immunoperoxidase staining of tissues using different antibodies after modified heat induced antigen retrieval. a) Staining of rat liver tissue using proliferating cell nuclear antigen antibody (invitrogen) 40X. b) Staining of rat thymus using anti-CD 45 antibody (Abcam) 40X. c) Staining of rat liver using CYP 3A antibody (Santa Cruz) 10X. d) Staining of rat kidney using KIM 1 HAVCR antibody (R&D sysytems) 10X. e) Staining of rat heart using anti-CD 45 antibody (Abcam) 40X.

Employing this method, we performed successful immunostaining for several tissues including adrenal, liver, thymus, kidney and heart without any damage or loss of section from the slide (Fig. 4). A quantitative comparison of tissue morphology after directly boiling in buffer with slide clipping and without slide clipping (Fig. 3) shows that this modified HIER method is undoubtedly helpful in immunohistochemistry staining of formalin fixed paraffin embedded tissues.

## Declaration of interest

The authors report no conflicts of interest. The authors alone are responsible for the content and writing of the paper.
